# Scientific

**Published:** 1889-06

**Authors:** 


### SCIENTIFIC ITEMS.

**The Anæsthetic revelation.**—Until I saw the article by Mr. J. H. Seymour in the March number of your journal, I had never heard of such an experience as "Anæsthetic Revelation," yet I was strangely moved on reading the above article, for, a few years ago, I myself had an experience which in its general features was very similar to the one detailed. Having previously tried gas, or what is advertised as "vegetable vapor," with very unsatisfactory results, and having eight badly decayed teeth to be extracted, I concluded, with the advice of my dentist, to try ether. My sensations in entering the state of complete insensibility were somewhat like those of your correspondents. I remember that long after all voluntary muscular power was gone, my sense of hearing seemed painfully acute. After several unsuccessful attempts with a rubber appliance over my face, I finally succumbed and remained insensible for about twenty minutes. My first remembrance of this interval is, that all seemed plunged in total darkness, my disembodied spirit seemed hung in the midst of primeval nothingness; mystery and despair filled my being. The main thought seemed to be that of dense ignorance as to the existence of God or of any plan concerning man's creation. From this feeling I began to reason that if there was a God I would not be left in such a fearful condition as I was then in. At this point it seemed clear to me that if I could bring myself honestly and unreservedly to agree to certain conditions, all would be made plain. Then came a struggle, the conditions seemed hard, but I felt that if they were requisite to the knowledge which I craved, they must be fulfilled. I agreed fully to the requirements, and the demonstration came. Words cannot express the sensations that filled my whole being. It would seem extravagant if I should undertake to tell the rapture which overflowed my soul; hopes which I had renounced as the conditions of enlightenment seemed to blossom into realities. Other conditions, which before were irksome, now teemed with joy. One of the latter conditions was that I should make it my life work to proclaim this "Revelation," and prove to mankind by indisputable evidence and logic the existence and care of an all wise God. I was eager to commence at once to proclaim my message. I realized that many would not receive the truth, but was not deterred by the thought. Just at this juncture I regained sensibility and felt the forceps upon the last of the eight teeth. So anxious was I to tell what had been revealed to me that I partially freed myself from the hold of the assistant. The operator urged me to remain quiet for a moment, which I did, and the tooth was removed. I then stepped from the chair, and turning to the dentist and my brother, who was assisting him, said solemnly, Mudge, I have had a revelation. There is a God and I am prepared to prove it. I felt that I could do so beyond any dispute and as clearly as any mathematical problem could be demonstrated, but as I came more and more to myself the clearness of the proof faded away, though the feeling that it had been possessed remained and still remains.—E. H. BLOOD in *Pop. Sc. News.*

**Sixty Miles to the Moon.**—The following is from the Boston *Herald* of recent date and is headed " Bragging to Some Purpose :"

"When some ten or twelve years ago the Lick Observatory of California ordered of Mr. Alvin Clark, of Cambridge, Mass., a thirty-four inch object glass for a telescope, the size seemed so preposterous that, taken in connection with the rather ominous name of the donor of the funds, it looked like a fair and square notice to the universe that California intended to lick the whole astronomical world, Washington, Greenwich, Paris and St. Petersburg included. What was more to the purpose, she actually did it. A glass of such power was constructed as to bring the moon within 200 miles of San Francisco, and so to furnish its citizens with a lunar line immeasurably shorter than could be boasted by any rival city. One would think that there were laurels enough for a State scarcely forty years old. But no, the glorious spirit of brag, finely developed as it may be on the Atlantic coast, swells to the sublimer proportions when it catches sight of the immeasurable Pacific. Everything in California has to be on the scale of the Pacific. The trees must be 400 feet high, the potatoes four to the bushel. Why not equally observatories? For some time, therefore, ardent spirits in the State have been chafing over the idea of having the moon as much as two hundred miles away. It has been making them feel so lonely that at last the discontent has found vent on a pecuniary scale so immense that the University of Southern California has been empowered to order of Mr. Clark a forty-two inch lens that will bring the moon within a distance of sixty miles, and so serve Mr. Lick himself with the same sauce with which he served the rest of the world. With five years to do it in, and $100,000 to oil the way, Mr. Clark says he can accomplish the feat."—*Pac. Med. Rec.*

**A Wonderful Photographic Feat.**—The New York *Tablet* publishes the following :

" Mr. Friese Green, a British photographer, has actually produced a picture with only the light issuing from his eye. Having stared for fifteen seconds at a 3,000 candle electric arc only three feet away, he closed his eye and quickly brought it upon a sensitive plate at a distance of one inch. The result was a very faint but distinct image of the arc and the carbons, due probably to momentary phosphorescence of the retina. A second attempt failed, and gaslight proved too weak to produce effect.—*Ib.*

**Microscopic** examination of cross-sections of horse hairs may reveal what are the peculiarities of the cortex which cause unequal changes when moistened, and thus produce writhing motions so similar to the muscular movements of the Gordius or hair-worm. So deceptive are these mechanical movements of the hairs, that one can readily pardon the belief that they are really transformed. Will not microscopists follow up the lines of inquiry herein suggested.—*Am. Micro. Journal.*

